# Digital Insights Into Nucleotide Metabolism and Antibiotic Treatment Failure

**DOI:** 10.3389/fdgth.2021.583468

**Published:** 2021-03-03

**Authors:** Allison J. Lopatkin, Jason H. Yang

**Affiliations:** ^1^Department of Biology, Barnard College, New York, NY, United States; ^2^Department of Ecology, Evolution, and Environmental Biology, Columbia University, New York, NY, United States; ^3^Data Science Institute, Columbia University, New York, NY, United States; ^4^Ruy V. Lourenço Center for Emerging and Re-emerging Pathogens, Rutgers New Jersey Medical School, Newark, NJ, United States; ^5^Department of Microbiology, Biochemistry and Molecular Genetics, Rutgers New Jersey Medical School, Newark, NJ, United States

**Keywords:** antibiotic resistance, antibiotic tolerance, antibiotic persistence, nucleotide metabolism, whole genome sequencing, machine learning, metabolic modeling, predictive modeling

## Abstract

Nucleotide metabolism plays a central role in bacterial physiology, producing the nucleic acids necessary for DNA replication and RNA transcription. Recent studies demonstrate that nucleotide metabolism also proactively contributes to antibiotic-induced lethality in bacterial pathogens and that disruptions to nucleotide metabolism contributes to antibiotic treatment failure in the clinic. As antimicrobial resistance continues to grow unchecked, new approaches are needed to study the molecular mechanisms responsible for antibiotic efficacy. Here we review emerging technologies poised to transform understanding into why antibiotics may fail in the clinic. We discuss how these technologies led to the discovery that nucleotide metabolism regulates antibiotic drug responses and why these are relevant to human infections. We highlight opportunities for how studies into nucleotide metabolism may enhance understanding of antibiotic failure mechanisms.

## Introduction

In the nearly 100 years since the discovery of penicillin, antibiotics have revolutionized medical practice and have become a cornerstone of modern medicine. However, growing rates of antimicrobial resistance pose an urgent and looming threat to public health and economic stability (1). These are compounded by a diminished antimicrobial discovery pipeline (2), creating a critical need to understand mechanisms responsible for antibiotic treatment failures and to discover new effective antimicrobials.

Clinical microbiology traditionally relies on general microbiology and molecular biology laboratory techniques, such as polymerase chain reaction and gene deletion/over-expression, to elucidate molecular mechanisms responsible for clinical phenotypes. However, experimental throughput by these methods limits progress toward understanding mechanisms of antibiotic treatment failure. In recent years several new experimental and digital technologies have emerged with promise to increase clinical microbiology laboratory throughput and enhance clinical management of bacterial infections (3–5). Moreover, advances in prokaryotic systems biology (6, 7) and interpretable machine learning (8) are for the first time accelerating discovery of mechanisms underlying antibiotic efficacy (9, 10).

Here, we review emerging digitalization technologies poised to transform research into mechanisms of antibiotic treatment failure in the clinic. We describe several antibiotic resistance, tolerance and persistence mechanisms discovered from clinical strains. We discuss in detail the recent discovery that nucleotide metabolism actively participates in antibiotic lethality and the clinical relevance of these findings (11). We propose new opportunities for digitalization technologies to advance clinical practice and to open frontiers for basic research into nucleotide metabolism and antibiotic efficacy.

## Digitalization in Clinical and Research Settings

The most important goal in clinical microbiology is to identify an infectious pathogen and determine its drug susceptibility profile (12). Traditionally, clinical microbiology laboratories rely on culture-based methods for pathogen identification and susceptibility testing. These approaches require the successful isolation and culture of pathogen cells from a clinical sample, followed by *in vitro* screening with standardized antibiotics.

*In vitro* studies in research settings have enabled the discovery of antibiotic resistance mechanisms. For example, following the initial detection of clinical tetracycline resistance, several microbiology studies identified decreased drug transport as the mechanism responsible for reduced efficacy (13, 14). Subsequent studies identified multi-drug resistant efflux pumps in multiple pathogenic species (e.g., AcrB in *Escherichia coli* and MexB in *Pseudomonas aeruginosa*) (15). As with their clinical counterparts, these fundamental studies rely on culture-based growth and targeted sequencing; however, such experimental technologies are resource- and labor-intensive and do not scale well with the plethora of pathogen variants, drug mechanisms, and resistance strategies found in the clinic.

In recent years, advances in laboratory evolution, high-throughput sequencing, and computational biology have greatly expanded the scope of addressable questions in microbiology and the study of antibiotic resistance (16). For instance, adaptive laboratory evolution can simulate natural selection pressures (17), allowing researchers to study the emergence of novel antibiotic treatment phenotypes (18), as well as their relationship to environmental conditions (19). In many cases, these granular experimental techniques invite complementary computational modeling activities, from mechanistically simulating drug-target binding to predicting complex ecological dynamics, yielding deeper insights into clinical resistance phenomena.

Concurrently, whole-genome sequencing has transformed the study of antibiotic resistance, enabling the identification of all possible gene variants that can give rise to clinical phenotypes (20). Whole-genome sequencing has proven instrumental in revealing population- and epidemiological-level insights into pathogen detection and emergence. For example, the 2011 outbreak of the Shiga-toxin producing enteroaggregative *E. coli* O104:H4 resulted in over 3,000 infections and more than 50 deaths – rapid, open-access whole-genome sequencing analysis revealed the phylogenetic relationships between this strain and 40 previously published pathogen genomes (21). These analyses conclusively demonstrated that O104:H4's virulence was attributable to the horizontal acquisition of *stx2*, along with other unexpected traits heretofore unseen in this lineage (22). Indeed, whole-genome sequencing enables insights into a pathogen's plasticity and facilitates real-time epidemiological tracing (23).

Whole-genome sequencing has spurred the development of advanced computational techniques capable of inferring meaningful biological relationships. Advances in mathematical modeling and machine learning are now, for the first time, enabling the direct identification of antibiotic resistance determinants from the genomes of clinical isolates in as *Staphylococcus aureus, P. aeruginosa*, and *E. coli* (24). Moreover, mathematical modeling and high-throughput sequencing approaches have revealed that sub-inhibitory selection and step-wise adaptation play just as important a role in antibiotic treatment failure as canonical antibiotic resistance mechanisms (25). Indeed, clinical isolates from patients with relapsed *Mycobacterium tuberculosis* infection exhibit sub-breakpoint minimum inhibitory concentrations (MICs) in comparison to strains from patients durably cured (26). Mutations responsible for such subtle cellular phenotypes are readily overlooked using previous methods. Additionally, machine learning can complement traditional culture-based methods and enable the direct prediction of pathogen MICs (27, 28) and provide experimentally testable insights into antibiotic mechanisms of action (9).

## Antibiotic Treatment Failure Mechanisms in Clinical Pathogens

Antibiotic treatment failure is conventionally understood to be fully explained by antibiotic resistance, in which a pathogen acquires a genetic mutation either to reduce the ability of an antibiotic to inhibit its target or reduce the effective intracellular concentration of an antibiotic (15, 29). Indeed, antibiotic resistance mutations from sequenced clinical isolates frequently appear in either the target of the antibiotic, modifying the ability of an antibiotic to bind, or in the promoter regions of drug efflux pumps, inducing antibiotic export (30). Other antibiotic resistance alleles, such as genes encoding β-lactamases, commonly appear in mobile genetic elements and can become exchanged by horizontal gene transfer (31).

However, in recent years there has been a growing recognition that alternative bacterial phenotypes, such as antibiotic tolerance (in which isogenic bacteria exhibit slower killing by an antibiotic) and antibiotic persistence (in which isogenic bacteria exhibit a shallower antibiotic killing plateau), also lead to treatment failure and relapsed infection (32). Additionally, there is growing appreciation that the local microenvironment of infection can act on several aspects of bacterial physiology to alter antibiotic treatment efficacy (33, 34). In fact, the local metabolic microenvironment of an infection is highly dynamic and local metabolites induced by either infection or antibiotic treatment itself can inhibit a pathogen's cellular response to antibiotic exposure (35).

**Figure 1:**
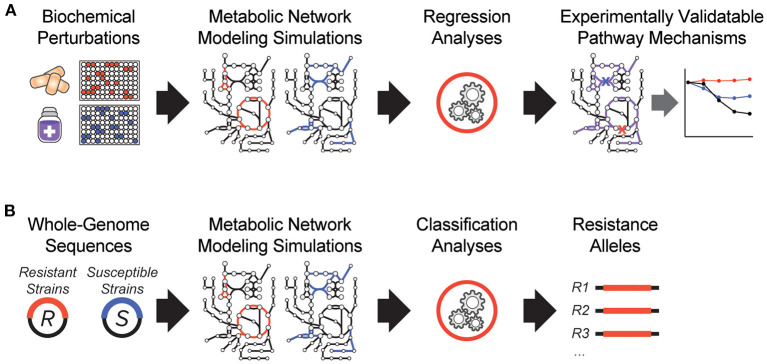
Recent innovations in interpretable machine learning for studying antibiotic treatment failure. **(A)** A biochemical screen was combined with metabolic network modeling and machine learning regression analyses to elucidate pathway mechanisms of antibiotic lethality. This led to the discovery that purine biosynthesis is a critical component of bactericidal antibiotic lethality (9). **(B)** Whole-genome sequencing data from antibiotic resistant (R) and susceptible (S) strains from clinical strains were applied as modeling constraints to genome-scale metabolic models. Machine learning classification analyses were applied (10).

It is clear that antibiotic-target interactions alone are insufficient for explaining antibiotic treatment failure in human patients. To address these knowledge gaps, interpretable machine learning approaches are being developed, which seek to rapidly generate experimentally testable hypotheses for biological phenomena. In one of the earliest demonstrations of these, a biochemical screen was performed to measure changes in antibiotic efficacy following metabolic stimulation, and genome-scale metabolic modeling simulations were performed to estimate metabolic reaction activities in each screening condition (Figure 1A). By applying machine learning to these data, purine biosynthesis was identified as a prominent player that governs antibiotic efficacy (9), highlighting a target-independent aspect of bacterial physiology is commonly involved in the lethal process of diverse bactericidal antibiotics. In light of the central role that purine metabolites also play in regulating the immune system (36), these results are also suggestive of mechanisms by which the patient-specific metabolic environment of an infection can promote drug tolerance or antibiotic treatment failure.

In another study, a metabolic model-based machine learning classifier was developed, which uses flux balance analysis to estimate the biochemical effects of genetic mutations characterized from clinical isolates (Figure 1B). Applying this approach to a large collection of genomes from drug-tested *M. tuberculosis* strains, novel metabolic resistance mechanisms to first-line tuberculosis antibiotics were discovered (10). These two examples illustrate how network models can serve as quantitative knowledgebases (37) and be combined with machine learning analyses to learn molecular mechanisms responsible for antibiotic treatment failures directly from clinical isolates (38).

## Nucleotide Metabolism in Antibiotic Treatment Failure

Bacterial metabolism is now understood to be an important physiological regulator of antibiotic efficacy (39). Across living systems, cellular metabolism is governed by the synthesis, allocation, and utilization of energy; and a growing number of studies demonstrate that metabolic dormancy protects cells from antibiotic treatment by inducing a phenotypically tolerant physiological state (29). Moreover, ATP synthesis correlates with the lethality of bactericidal antibiotics better than bacterial growth rates (40), suggesting that antibiotic-induced lethality is an active process and not merely a passive consequence of the loss-of-function of an essential gene product.

In particular, bactericidal antibiotics have been shown to elevate central carbon metabolism activity (41, 42) and trigger the formation of byproduct reactive oxygen species (43, 44), which damage DNA and cause bacterial lethality (45–47). These phenomena are not restricted to antibiotics, as reactive oxygen species also actively contribute to the lethality of bacterial secretase dysfunction (48) and thymine depletion (49). Moreover, defects in central carbon metabolism activity are linked to antibiotic tolerance and persistence across many bacterial species (50–53) and can be stimulated to enhance antibiotic efficacy (54, 55). However, antibiotic treatment perturbs several aspects of bacterial metabolism beyond central carbon metabolism (56), highlighting important knowledge gaps in understanding how different metabolic pathways may contribute to antibiotic treatment failure.

It may come to no surprise that nucleotide metabolism is actively involved in antibiotic efficacy (9). Nucleotides are essential metabolites and are ubiquitous to all living cells; in addition to their roles as fundamental building blocks for DNA and RNA molecules, constituting more than 20% of cellular biomass (57), nucleobases also form the molecular basis of primary energy currencies such as ATP and NADH, and many coenzymes are derived from nucleobase monomers. In fact, the thermodynamic properties of nucleobases are so special, that these metabolites synchronize cell biochemistry and regulate biochemical group transfers across diverse physiological processes (58). Moreover, the concentration of intracellular ATP is tightly regulated across the tree of life and heavily buffered across environmental conditions (59).

**Figure 2:**
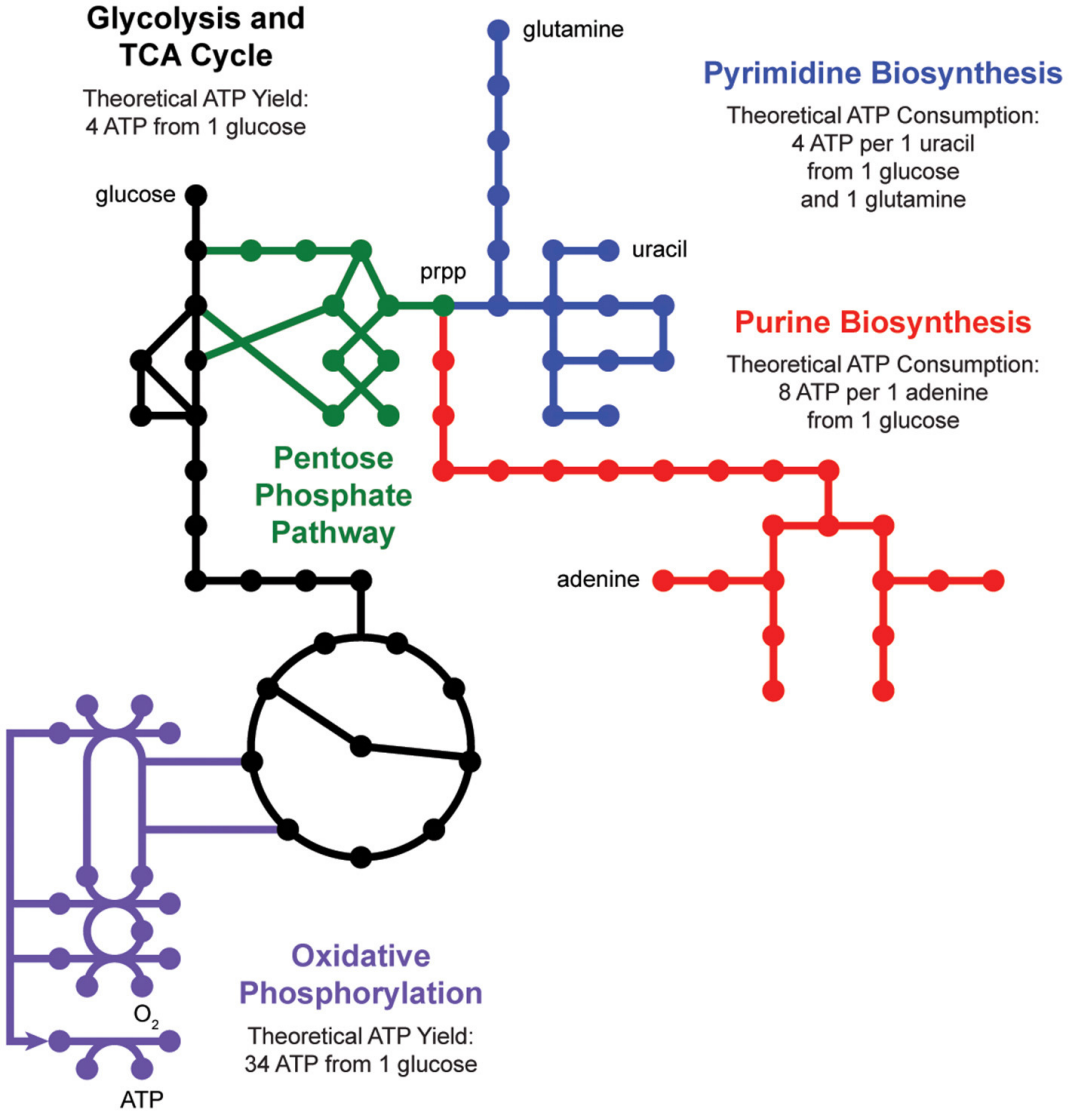
Nucleotide metabolism is energetically expensive. *De novo* biosynthesis of purines (red) and pyrimidines (blue) begins with the pentose phosphate pathway (green), which generates phosphoribosyl pyrophosphate (prpp) from glycolysis (black). The energetic demand for ATP molecules to power purine and pyrimidine biosynthesis drives activity through the tricarboxylic acid (TCA) cycle (black) and oxidative phosphorylation (purple).

*De novo* nucleotide biosynthesis from carbohydrates begins with the pentose phosphate pathway, which supplies phosphoribosyl pyrophosphate (prpp) as a shared substrate to the purine and pyrimidine biosynthesis pathways (Figure 2). These pathways produce nucleotide triphosphates which can be incorporated into DNA and RNA or processed into energy currencies that can power virtually all other biochemical processes in the cell. Interestingly, nucleotide biosynthesis is itself an energetically demanding process, costing a cell 8 ATP molecules to synthesize one adenine molecule from one glucose molecule. Indeed, cells employ a multitude of strategies to manage these tradeoffs, including prioritized nutrient usage, maintenance metabolism, and nucleotide salvage.

Antibiotic treatment imposes additional layers of complexity on these processes; cells must expend energy to mount defensive stress responses, and many antibiotics preferentially kill metabolically active cells. Specific components of nucleotide metabolism have been shown to contribute to antibiotic efficacy and protection both *in vitro* and *in vivo*. In many cases, defects in nucleotide biosynthesis have been shown to induce antibiotic persistence, suggesting these may represent a key metabolic strategy for evading antibiotic efficacy. For example, several chemogenomic screens identify nucleotide biosynthesis genes, as well as global regulators of nucleotide metabolism, as important regulators of antibiotic tolerance (60, 61). Likewise, antibiotic drug screening under nutrient limitation identified several compounds that interfere in core or peripheral nucleotide metabolism branching points (62).

Of note, purine biosynthesis frequently emerges as a key pathway responsible for antibiotic efficacy. For example, in an antibiotic persistence screen using a *S. aureus* transposon mutant library, 29% of all depleted genes were related to cellular metabolism, and of these, five were involved in purine biosynthesis (63). These *ex vivo* observations are important for understanding clinical antibiotic treatment failure, as methicillin-resistant *S. aureus* clones isolated from patients enduring multi-drug antibiotic treatment were found to possess mutations in *purR*, a transcriptional repressor of purine synthesis, within 1 week of treatment. *In vitro* follow-up experiments confirmed that this mutation reduced the rate of vancomycin-induced killing, revealing the evolution of antibiotic tolerance *in vivo* (64). Importantly, this mutation preceded the onset of canonical resistance evolution; these and other studies suggest that mutations in nucleotide metabolism may help create a reservoir of pathogen cells primed to subsequently evolve target-specific antibiotic resistance alleles.

**Figure 3:**
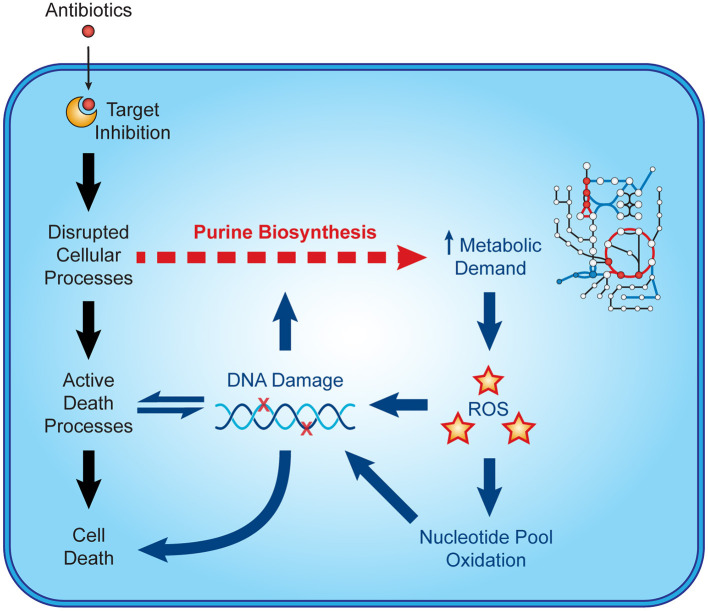
Nucleotide metabolism contributes to antibiotic lethality. In addition to their target-specific effects, bactericidal antibiotics induce purine biosynthesis, which increases activity in central metabolism. Increases in central metabolism stimulate the production of toxic reactive oxygen species, which oxidize nucleotides and damage DNA. These insults to DNA and the nucleotide pool induce bacterial death and may further potentiate purine biosynthesis.

Recent microbiological studies are beginning to clarify how nucleotide metabolism contributes to antibiotic efficacy (Figure 3). Interpretable machine learning analyses reveals that several metabolic pathways proximal to purine biosynthesis contribute to the lethality of bactericidal antibiotics in *E. coli* (9). Purine biosynthesis becomes induced by bactericidal stress-induced adenine limitation, which can be directly measured by targeted metabolomics (56). Consequently, oxidative phosphorylation becomes elevated to meet the increased energetic demand of enhanced purine biosynthesis, increasing cellular respiration and central carbon metabolism and providing substrates for toxic reactive oxygen species (42, 43). Indeed, regulation of nucleotide metabolism appears to be a well-conserved mechanism that bacteria have evolved to handle diverse stresses (65).

Consistent with these, purine nucleotides such as (p)ppGpp function as universal alarmones for transcriptionally activating the stringent response and other bacterial stress responses as evolutionally conserved strategies for surviving nutrient limitation and other environmental stressors (66, 67). Intracellular accumulation of (p)ppGpp and related purine alarmones can induce antibiotic tolerance by promoting growth arrest (68) and entry to antibiotic persister states (69). Recent studies demonstrate that in additional to these transcriptionally mediated programs, (p)ppGpp can also inhibit nucleotide metabolism directly by binding several enzymes involved in purine biosynthesis, including PurF and Gsk (70, 71). These data collectively support a central role for nucleotide metabolism in antibiotic treatment efficacy.

It is interesting to note that nucleotide metabolism is also very important for the *in vivo* pathogenesis of diverse bacterial infections, and may be required for a pathogen's growth and survival within the host environment (72). For instance, *S. aureus* cells with transposon insertions in *purB* fail to establish bone infections in mice (73) and deletion of purine biosynthesis genes prevents uropathogenic *E. coli* from expanding into intracellular bladder epithelial cells (74). Likewise, *in vivo* studies of methicillin-resistant *S. aureus* showed that purine biosynthesis was causally linked to survival during endovascular infection (11). Collectively, it is clear that nucleotide metabolism, particularly purine biosynthesis, plays an important role in bacterial pathogenesis and in the response to antibiotic stress.

## Discussion

The growing challenge of clinical antibiotic failure demands renewed attention into the study of antibiotic mechanisms of action and the discovery of new antimicrobial compounds. Digital technologies such as whole-genome sequencing, machine learning, mass spectrometry and predictive modeling are likely to transform the clinical management of bacterial infections in the coming decades. Exciting developments in machine learning are, for the first time, enabling the rapid discovery of novel classes of antimicrobial compounds (75) and the rapid identification of bacterial pathogens in the clinic (5). Advances in mass spectrometry-based metabolomics are enabling the rapid discovery of antimicrobial mechanisms of action (76). Advances in predictive modeling (7) are enabling new understanding into the complex ecology of microbial communities (77).

The discovery that nucleotide metabolism is involved in antibiotic efficacy has several translational implications. Unlike the Mueller-Hinton or Luria-Bertani media commonly used by clinical and academic microbiology laboratories, the metabolic microenvironment of a bacterial infection is dynamically enriched for nucleotide metabolites during infection (35). In fact, purine metabolites are important regulators of innate immunity (36), playing dual roles in regulating the host response to infection and the pathogen response to antibiotics. Nucleotide analogs are also commonly used to treat human cancers and viral infections and have potential to address antimicrobial resistance in the clinic (78, 79).

Nucleotide metabolism is one of the oldest areas of bacterial physiology to be investigated, with early studies into bacterial purine and pyrimidine metabolism predating the discovery of the lac operon (80, 81). Interest in nucleotide metabolism is mounting a resurgence, spurred by the growing recognition that nucleotides play important roles in both immunometabolism (82, 83) and cancer pathogenesis (84). Given that purine and pyrimidines exert opposing effects on antibiotic efficacy and carbon metabolism in bacteria (9), nucleotide metabolism represents an exciting open frontier for future studies in bacterial physiology and antibiotic treatment failure.

Concurrently, new digitalization techniques are becoming increasingly democratized and are poised to transform our basic and translational understanding of how nucleotide metabolism may contribute to antibiotic efficacy. Advances in predictive modeling (7) and non-targeted metabolomics (85) are revealing the diverse systems-level consequences of antibiotic stress. Quantitative microscopy advances (86) are enabling detection of antibiotic tolerance and resistance at single-cell resolution. Advances in transposon insertion sequencing (87) and adaptive lab evolution (88) are revealing new mechanisms for antibiotic resistance. Indeed, it would be exciting for future discoveries to reveal how nucleotide metabolism may contribute to antibiotic failure mechanisms beyond persistence (11) and potentially rewrite our understanding of antimicrobial resistance (29).

## Author Contributions

AL and JY planned, wrote, and edited the manuscript. All authors critically read, reviewed, and approved the final version of the manuscript.

## Conflict of Interest

The authors declare that the research was conducted in the absence of any commercial or financial relationships that could be construed as a potential conflict of interest.
